# Examining tactical sprint actions and distribution among playing positions attending to match status in soccer: Implications for specific training

**DOI:** 10.1371/journal.pone.0301925

**Published:** 2024-06-10

**Authors:** David Lobo-Triviño, Tomás García-Calvo, David Manzano-Rodríguez, Fabio Nevado, Marcos Chena, Juan Ángel Piñero-Madrona, Emilio Martín-Ardila, Javier Raya-González

**Affiliations:** 1 Faculty of Sport Sciences, University of Extremadura, Cáceres, Spain; 2 LaLiga Sport Research Section, Madrid, Spain; 3 Spanish Association of Physical Trainers, Madrid, Spain; Università degli Studi di Milano: Universita degli Studi di Milano, ITALY

## Abstract

The study aimed to analyze the tactical sprint actions performed by Spanish professional soccer players, considering their playing positions and the match status at the time of each action. Thirty-two Spanish male professional soccer players from a LaLiga Spanish Second Division (LaLiga SmarthBank) team participated in this study. Actions above 85% of the players’ maximum velocity during 42 official matches were collected by an optical tracking system ChyronHego® and were synchronized using Mediacoach software (LaLiga, Madrid, Spain). Then, actios were analyzed trough an observation instrument designed to assess the type of tactical action performed by players. Central defenders (CD) and wide defenders (WD) were mainly involved in recovery runs. Central midfielders (CM) also frequently performed recovery runs and pressing actions. Wide midfielders (WM) were often engaged in runs in behind/penetrate actions, while forwards (F) had a diverse range of sprint actions, including pressing, runs in behind/penetrate, and breaking into the box. It was observed that F performed fewer chase actions than expected. On the other hand, CD, WD, and CM engaged in a greater number of recovery run actions than expected. CD also performed more close down/interception actions than expected, while CM, WM, and F performed fewer close down/interception actions than expected. When their team was losing, WM performed more recovery run actions than expected. CM made more runs with the ball when their team was winning CD showed a higher frequency of breaks into the box when their team was winning. These findings provide valuable information regarding the tactical aspects of sprinting in soccer, facilitating the design of specific training tasks that not only address the physical demands associated with each playing position but also considering the tactical context in which sprints occur.

## Introduction

Soccer is a highly demanding team-sport characterized by the presence of a great number of high-intensity actions (HIA) such as jumps, accelerations, decelerations and sprints [1]. Specifically, sprint demands have increased in recent years [2], with professional soccer players often required to cover sprint actions longer than 30 m and to reach speeds of about 30 km·h^-1^ during their execution [3, 4]. Also, sprint actions may be considered a prerequisite for successful performance in soccer, since they are associated with critical actions such as scoring a goal [5], creating a shot, or evading an opponent [6]. For these reasons, strength and conditioning coaches must design effective training drills to improve sprinting skills to enhance performance, increase player availability, and improve rehab programs [4].

For a better understanding of sprinting during match-play, it is essential to consider contextual variables, as highlighted in the study conducted by Raya-González et al., [7]. Previous research has shown that physical demands during matches are influenced by playing positions, particularly in terms of high-intensity running and sprinting distance [8]. For example, Oliva-Lozano et al., [4] found that central defenders (CD) cover shorter sprint distances compared to central midfielder (CM) and wide midfielder (WM). Therefore, when designing individualized training programs, it is essential to consider the specific playing position of each player. Additionally, match outcomes have been found to have a significant impact on match-play demands. Augusto et al. [9] observed that in matches resulting in losses, high-intensity running and acceleration were higher compared to draws and wins. Similarly, Chmura et al. [10] revealed that full-backs, central defenders and central midfielders covered significantly shorter distances at certain intensities (i.e., 17–20.99 and 21–23.99 km·h^-1^) in matches they won. Despite their promising results, the aforementioned studies were focused on the final match outcome. Thus, a more comprehensive analysis, including a classification of each sprint based on the match temporal status at the moment the action occurs and considering the differentiation among playing positions is required. This information provides to practitioners more detailed and specific information to design training strategies and optimize performance for individual players and the team.

Although physical metrics provide valuable insight to practitioners, this data seems to have limited application for training purposes because it is unknown how and why players perform those actions [8, 11]. Therefore, tactical information about sprint actions in professional soccer is necessary. To date, only a small number of studies have been conducted for this purpose. For example, Ju et al. [8] conducted a tactical analysis of the sprint actions performed by English Premier League teams during a whole season, focusing on playing positions. These authors observed that WM covered the greatest distance sprinting when run with ball, whereas wide defenders (WD) performed more over/underlap distance compared to other playing positions. Additionally, CD and WD completed more distance performing high-intensity covers than other playing positions. Similarly, Oliva-Lozano et al. [4] examined the effect of playing position on physical and tactical sprint demands in Spanish professional soccer players. In this regard, the authors observed that WM performed fewer maximal intensity sprints without the ball than expected, while CD performed more sprints with the ball than expected. These authors also showed that the frequency of tactical sprint actions is influenced by playing positions, with interceptions being the most frequently performed actions by CD. On the other hand, WC, WD and FW usually sprinted to run the channel to receive/exploit space, while MC completed most of their sprints for recovery runs. Despite this, the aforementioned studies did not consider the number of actions performed per playing position or the match status, which is necessary to identify specific sprint patterns and replicate it in order to optimize the training process for each playing position.

Based on the identified gaps, contextualized studies focusing on the tactical demands of sprinting, considering playing positions and the match result at the moment when these actions occur are required. Therefore, the aim of this study was to provide a contextual analysis of the sprinting tactical demands among Spanish professional soccer players, considering their playing positions and the match status at the time of each action. This analysis aims to aid in the development of targeted training tasks for improving sprint performance.

## Method

### Participants

Thirty-two professional male soccer players (age: 27.2 ± 5.6 years, height: 182.3 ± 4.2 cm, body weight: 78.5 ± 5.5 kg) were included in the study. These players belonged to the same team and competed in the 20/21 season of LaLiga Spanish Second Division (LaLiga SmarthBank). The team consisted of 6 CD, 3 WD, 11 CM, 8 WM and 4 F. Goalkeepers were excluded for the analysis due to the different nature of their activity profile. For the analysis, all actions in which the players achieved a maximum speed higher than 85% of their reported maximum speed during the season (i.e., 42 official matches) were selected [12]. A total of 1853 actions were initially selected, and after excluding 5 actions that were not related to any tactical-technical aspect of the game, a final sample of 1848 actions was obtained. The data used in the study were provided by LaLiga^TM^, which had informed all participants through its protocols. Written informed consent was obtained from all the participants and from a parent and/or legal guardian for subjects under 18. The data were accessed at the end of the 22/23 season, specifically on June 20, 2023. All data were anonymized according to the Declaration of Helsinki to ensure players’ and teams’ confidentiality, and the protocol followed the ethical standards for studies in Sports Sciences.

### Design and procedures

A retrospective, descriptive longitudinal design was applied to analyze the sprinting tactical demands among Spanish professional soccer players, considering their playing positions and the match status at the time of each action. All the sprint actions performed by soccer players were analyzed, and an observation instrument based on the integrated approach of Ju et al. [13] was designed to assess the type of tactical action performed by players. The actions were categorized according to the classification presented in Table 1.

**Table 1:** Descriptions of the variables within the integrated approach.

Actions	Description
***Defense***	
*Chase*	Player chases an opponent who has the ball.
*Press*	Player runs directly towards opposition player on/or receiving the ball, or towards space or players not on/receiving the ball (typically blocking passing channels).
*Recovery run*	Player runs back towards their own goal to be goal side when out of position.
*Close Down/Interception*	Player cuts out pass.
*Collective run*	Most of the players on the team move in the same direction simultaneously
***Attack***	
*Run with ball*	Player moves with the ball either dribbling with small touches or running at speed with fewer ball touches.
*Run in behind/Penetrate*	Player attacks space behind, overtakes and/or unbalances the opposition defense (typically ball is behind).
*Break into the box*	Player enters the opposition’s penalty box in an attempt to receive the ball (typically from a cross–ball in front and wide).
*Over/Underlap*	Player runs from behind to in front of the player on the ball or receiving the ball.
*Move to receive/Exploit the space*	Player moves to receive a pass from a teammate or to create/exploit space (typically comes short or moves wide to receive ball).

Furthermore, playing position and a contextual variable such as match status were included in the analysis. Specifically, the players were divided into five positions on the field: central defender (CD), wide defender (WD), central midfielder (CM), wide midfielder (WM) and forward (F). Finally, match status was recorded as “losing” (L), “drawing” (D) or “winning” (W), based on the score at the time the action was performed.

After the actions were collected based on the specified criteria, they were independently analyzed by four observers who were members of professional soccer staff and had experience in soccer research and video analysis. The observers underwent training on the interpretation of these types of actions, and they demonstrated almost perfect inter-rater reliability (Kappa statistic, κ = 0.85) and an almost perfect intra-rater reliability (Kappa statistic, κ = 0.90) [14]. The observers categorized the actions according to the possibilities presented in Table 1, and they reached a final agreement on 97% of the actions.

Match data were collected by an optical tracking system ChyronHego® (TRACAB, New York, US). This multi-camera tracking system consists in 8 super 4K-High Dynamic Range cameras based on a positioning system (Tracab—ChyronHego VTS) that records and analyzes X and Y positions for each player from several angles, thus providing real-time two-dimensional tracking (tracking data are recorded at 25 Hz). The system was used to identify actions that exceeded 85% of each player’s maximum speed. Additionally, a customized report was created using Mediacoach software (LaLiga, Madrid, Spain), which synchronized tracking data with the video footage of each match.

### Statistical analysis

A cross-table analysis was conducted to examine the distribution and relationship between tactical sprint actions and playing positions. The cross-tabulations visually and quantitatively represented the relationship between the categorical variables that were studied. The tactical sprint actions were arranged in rows, and the playing positions were arranged in columns. Each cell in the table represented the count of cases that belonged to a specific combination of tactical sprint action and playing position categories. The interpretation of the cross-tabulations was based on the comparison of the observed frequencies with those expected under the null hypothesis of independence between variables. This measure adjusts the magnitude of the residual as a function of the sample size and provides a way of evaluating the discrepancy between the observed values and the expected values. Furthermore, the analysis included the match status variable to examine the distribution and association of variables based on the score of the match. This additional factor allowed for a comprehensive examination of the variables in relation to the match status. Significance was considered when corrected residual values are not between -2 and 2. The data analysis was carried out using the Statistical Package for Social Sciences (SPSS 25.0; SPSS Inc., Chicago, IL, USA).

## Results

The distribution of tactical sprint actions varied among different playing positions. Among CD and WD, the most common tactical sprint actions were recovery runs, accounting for 42.49% and 35.06% of their total sprint actions, respectively. They were followed by close down/interception (32.08% for CD and 16.09% for WD) and chase (15.03% for CD and 14.66% for WD). For CM, the most frequent tactical sprint action was also the recovery run, accounting for 29.67% of their total sprint actions. It was followed by press (17.46%) and chase (15.07%). WM most performed run in behind/penetrate actions (19.95%), followed by press (15.52%) and a both chase and recovery run (13.30%). For F, the most frequent tactical sprint actions were press (27.85%), run in behind/penetrate (25.63%), and break into the box (18.67%).

Table 2 displays the distribution of actions performed according to playing positions. Significant differences were observed in several actions compared to the expected values. F performed significantly fewer chase actions than expected (error = -5.7). CD and WD performed a smaller number of presses than expected (error = -5.2 and -5.7, respectively), while F made more presses than expected (error = 7.4). CD, WD, and CM performed a greater number of recovery run actions than expected (error = 8.5, 5, and 2.6, respectively), while WD and forwards made fewer recovery runs than expected (error = -6 and -10.1, respectively). CD performed a greater number of close down/interception actions than expected (11.4 error), while CM, WM, and F made fewer close down/interception actions than expected (error = -5.2, -4.3, and -3, respectively). CD performed a smaller number of collective runs than expected (error = -3.8), while CM made more collective runs than expected (error = 2.5). CD performed a smaller number of runs with the ball than expected (error = -4.7), while WD and wide midfielders made more runs with the ball than expected (error = 4 and 3.6, respectively). CD, WD, and CM performed a smaller number of runs in behind/penetrate actions than expected (error = -6.8, -5.5, and -3, respectively), while wide midfielders and forwards made more runs in behind/penetrate actions than expected (error = 6.6 and 8.7, respectively).

**Table 2:** Cross-table for sprints registered (number) in match play considering each tactical purpose and playing position.

		CD	WD	CM	WM	F	Total
*Chase*	Count	52	51	63	54	10	230
	Expected count	42.9	43.3	52.1	50.5	41.1	230
	Corrected residual	1.6	1.4	1.8	0.6	-5.7	
*Press*	Count	18	15	73	63	88	257
	Expected count	48	48.4	58.3	56.4	45.9	257
	Corrected residual	-5.2	-5.7	2.4	1.1	7.4	
*Recovery run*	Count	147	122	124	54	10	457
	Expected count	85.3	86.1	103.6	100.4	81.6	457
	Corrected residual	8.5	5	2.6	-6	-10.1	
*Close Down/Interception*	Count	111	56	24	28	27	246
	Expected count	45.9	46.3	55.8	54	43.9	246
	Corrected residual	11.4	1.7	-5.2	-4.3	-3	
*Collective run*	Count	12	21	49	41	38	161
	Expected count	30.1	30.3	36.5	35.4	28.8	161
	Corrected residual	-3.8	-2	2.5	1.1	2	
*Run with ball*	Count	3	39	20	42	16	120
	Expected count	22.4	22.6	27.2	26.4	21.4	120
	Corrected residual	- 4.7	4	-1.6	3.6	-1.3	
*Run in behind/Penetrate*	Count	2	9	29	81	81	202
	Expected count	37.7	38	45.8	44.4	36.1	202
	Corrected residual	-6.8	-5.5	-3	6.6	8.7	
*Break into the box*	Count	1	6	32	42	59	140
	Expected count	26.1	26.4	31.7	30.8	25	140
	Corrected residual	-5.7	-4.6	0.1	2.4	7.8	
*Over/Underlap*	Count	0	29	0	0	1	30
	Expected count	5.6	5.7	6.8	6.6	5.4	30
	Corrected residual	-2.6	11	-3	-2.9	-2.1	
*Move to receive/Exploit space*	Count	0	0	4	1	0	5
	Expected count	0.9	0.9	1.1	1.1	0.9	5
	Corrected residual	-1.1	-1.1	3.1	-0.1	-1	

*Note*. CD = central defender; WD = wide defender; CM = central midfielder; WM = wide midfielder; F = forward.

**Fig 1:**
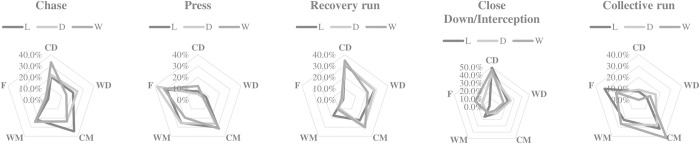
Distribution of defensive actions according to the match status and playing position; L = losing; D = drawing; W = winning; CD = central defender; WD = wide defender; CM = central midfielder; WM = wide midfielder; F = forward.

**Fig 2:**
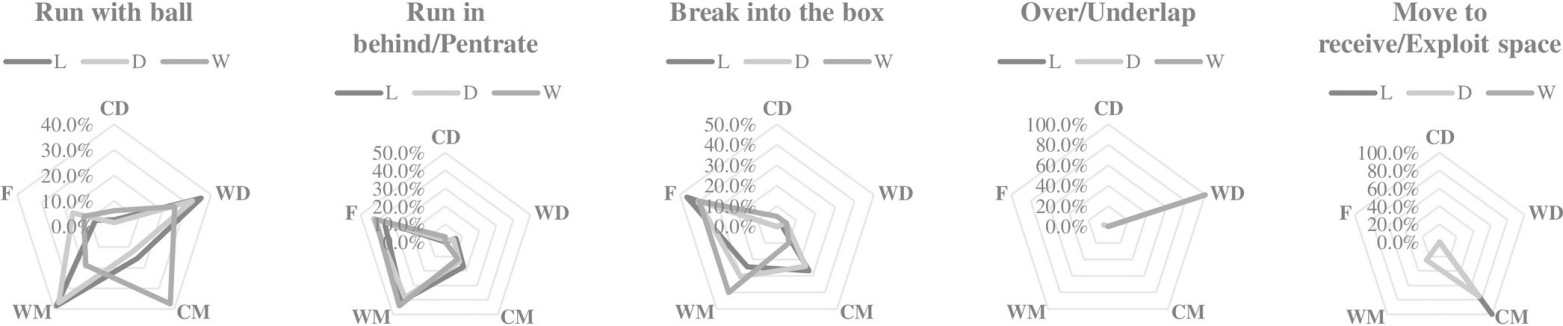
Distribution of attack actions according to the match status and playing position; L = losing; D = drawing; W = winning; CD = central defender; WD = wide defender; CM = central midfielder; WM = wide midfielder; F = forward.

Figs 1 and 2 shows the distribution of actions performed according to playing position and match status. When their team is losing, WM performed a greater number of recovery run actions than expected (error = 2.4), while when their team is drawing, they made a smaller number of recovery run actions than expected (error = -2.0). When their team is losing, F performed a smaller number of close down/interception actions than expected (error = -2.6), although when their team is winning, they made more actions of close down/interception than expected (error = 2.1). When their team was winning, CM performed a greater number of runs with the ball than expected (error = 2.4), meanwhile CD made a greater number of breaks into the box than expected (error = 2.5 error).

## Discussion

This study aimed to analyze the sprinting tactical demands in Spanish professional soccer players, considering their playing positions and the match status during each action. The unique aspect of this study lies in the combined quantitative and qualitative analysis of sprint actions, considering the specific positions of players and the match status at the time of each action. The results indicate that the frequency of tactical sprint actions differs across playing positions, and the status of the match influences the occurrence of these actions, particularly for WM and F.

The distribution of tactical sprint actions varies among playing positions in soccer matches, reflecting the different tactical roles they fulfill [15]. Defensive positions such as CD and WD, commonly engage in recovery runs, close down/interception, and chasing actions. CD players have a higher overall percentage of these actions, while WD players are also involved in running with the ball due to their field position. CM, exhibit similar frequencies, with defensive responsibilities and a significant number of sprints for pressing purposes. These findings partially align with prior study conducted by Oliva-Lozano et al. [4]. However, it is worth noting that there may be slight differences in the results, which could be attributed to the relative maximum velocity utilized in our study. Specifically, we employed a relative maximum velocity of 85% of the players’ maximum speed. This variance in methodology could contribute to the disparities observed in the frequency and type of sprint actions reported between the studies. In contrast, WM and F, despite their attacking roles, engage in sprint actions such as running in behind/penetrating and breaking into the box. These positions also undertake defensive actions, initiating pressing and contributing to actions such as pressing, chasing, and recovery runs. These obtained results show that the players sprint for different tactical purposes but that their playing position strongly influences the frequency of sprints, so training task must be designed accordingly to each specific playing position.

A cross-table analysis was conducted to assess whether playing positions completed the expected number of tactical sprint actions in each category. Regarding defensive actions, F performed fewer chase, recovery runs and close down/interception actions than expected, like WM in the latter categories and CM in the close down/interception category. Conversely, CD and WD performed more recovery actions than expected, and CD and CM completed more close down/interception actions than expected. In terms of pressing actions, F and CM completed more actions, while CD and WD performed a lower number of actions than expected. These findings support the results reported by Oliva-Lozano et al. [4], indicating that each playing position is influenced by certain patterns that shape their tactical sprint actions. Additionally, in terms of collective runs, CM completed more actions and CD fewer than expected. Run with ball actions were more frequently performed than expected by WD and WM, while CD completed a lower number of these actions. These patterns could be attributed to the specific game model adopted by the analyzed team, which focused their attacking actions on the side zones of the field [16]. The higher frequency of run in behind/penetrate and break into the box actions by forwards and wide midfielders aligns with their positional roles near the opponent’s goal, while these actions were less prominent for CD, WD, and CM. Lastly, wide defenders excelled in over/underlap actions, and central midfielders showed a tendency for move to receive/exploit space, reflecting the specific actions associated with each playing position [16]. These observed patterns underscore the influence of positional tendencies and strategic behaviors on the tactical demands of soccer, which must be considered when training sessions are designed, so that players can train specifically and cope with their match demands.

The analysis of the distribution of actions performed according to playing position and match status revealed several interesting patterns. When their team is losing, WM displayed a higher frequency of recovery run actions than expected, indicating their increased effort to regain possession and contribute to a potential comeback. However, when their team is drawing, WM exhibited a reduced number of recovery run actions, suggesting a more conservative approach to maintain the current result [16]. For F, when their team is losing, they performed fewer close down/interception actions than expected, potentially prioritizing their attacking contributions to equalize the match. Conversely, when their team is winning, F made more close down/interception actions than expected, highlighting their increased defensive efforts to protect the lead [15]. On the other hand, CM showed a higher number of runs with the ball than expected when their team is winning, indicating their active involvement in carrying the ball forward and creating attacking opportunities [16]. When their team is winning, CD demonstrated a greater number of breaks into the box than expected, although the overall frequency of these actions under this match status was limited (i.e., only 1 action was registered). These findings highlight the tactical variations and decision-making processes of players based on the match status. It indicates that different positions adapt their actions according to the game context, either focusing on defensive efforts, ball possession, or attacking movements attending to the specific necessities [15]. Strength and conditioning coaches could consider this information for a more comprehensive design of training sessions that address both the physical and tactical demands of the match-play. Also, it is recommended to consider the outcome of the prior match in order to adequately periodize the workload and recovery during the subsequent microcycle.

This study is not exempt of limitations. Firstly, only male soccer players were analyzed, so it is not possible to generalize to women’s soccer. Secondly, a single team was studied, which have their specific characteristics, such as formation, style, or strategy. Thus, further studies involving more teams are required, although larger sample size even though this is not easy in the context of professional soccer. Finally, match-related contextual variables such as team formation, team level, style of play or players’ age were not considered.

## Conclusions

This study confirms that the distribution of tactical sprint actions varied across different playing positions in professional soccer. CD and WD were commonly engaged in recovery runs, while CM frequently performed recovery runs and presented a higher involvement in pressing actions. WM were frequently involved in runs in behind/penetrate actions, while F had a diverse variety of sprint actions including pressing, runs in behind/penetrate, and breaking into the box. Also, F performed fewer chase actions than expected, while CD, WD, and CM covered a greater number of recovery run actions than expected. CD also performed more close down/interception actions than expected, while CM, WM, and F performed fewer close down/interception actions than expected. Additionally, WM performed more recovery run actions than expected when their team was losing, and CM made more runs with the ball while their team was winning. CD showed a higher frequency of breaks into the box when their team was winning. These findings provide valuable information related to how different positions adapt their actions and contribute to the team’s performance in various match scenarios. In a practical approach, strength and conditioning coaches must focus their efforts on designing specific training drills considering not only the physical demands associated with each playing position but also considering the tactical context in which sprints occur. Also, it is recommended to consider the outcome of the prior match in order to adequately periodize the workload and recovery during the subsequent microcycle.
